# The incidence and impact of atrial fibrillation on hospitalized Coronavirus disease‐2019 patients

**DOI:** 10.1002/clc.24240

**Published:** 2024-02-25

**Authors:** Haiming Niu, Jianwei Li, Catherine Teng, Xiaojia Lu, Chengyue Jin, Peng Cai, Ao Shi, Xiaoqing Shen, Qiqi Chen, Miaolian Chen, Yong Yuan, Pengyang Li

**Affiliations:** ^1^ Department of Critical Care Medicine Zhongshan People's Hospital Zhongshan P.R. China; ^2^ Division of Cardiology, Department of Medicine University of Texas Health Science Center San Antonio Texas USA; ^3^ Department of Cardiology, Mount Sinai Beth Israel Icahn School of Medicine at Mount Sinai New York New York USA; ^4^ Department of Cardiology the First Affiliated Hospital of Shantou University Medical College Shantou China; ^5^ Department of Mathematical Sciences Worcester Polytechnic Institute Worcester Massachusetts USA; ^6^ Faculty of Medicine St. George University of London London UK; ^7^ Department of Cardiovasculogy Zhongshan People's Hospital Zhongshan P.R. China; ^8^ Division of Cardiology, Pauley Heart Center Virginia Commonwealth University Richmond Virginia USA

**Keywords:** atrial fibrillation, COVID‐19, in‐hospital complication, mortality

## Abstract

**Background:**

Since 2019, Coronavirus disease‐2019 (COVID‐19) has raised unprecedented global health crisis. The incidence and impact of atrial fibrillation (AF) on patients with COVID‐19 remain unclearly defined.

**Methods:**

We conducted a retrospective cohort study using ICD‐10 codes to identify patients with a primary diagnosis of COVID‐19 with or without AF in National Inpatient Sample Database 2020. We compared the outcome of COVID‐19 patients with a concurrent diagnosis of AF with those without.

**Hypothesis:**

AF will adversely affect the prognosis of hospitalized COVID‐19 patients.

**Results:**

A total of 211 619 patients with a primary diagnosis of COVID‐19 were identified. Among these patients, 31 923 (15.08%) had a secondary diagnosis of AF. Before propensity score matching, COVID‐AF cohort was older (75.8 vs. 62.2‐year‐old, *p* < .001) and had more men (57.5% vs. 52.0%, *p* < .001). It is associated with more comorbidities, mainly including diabetes mellitus (43.7% vs. 39.9%, *p* < .001), hyperlipidemia (54.6% vs. 39.8%, *p* < .001), chronic kidney disease (34.5% vs. 17.0%, *p* < .001), coronary artery disease (35.3% vs. 14.4%, *p* < .001), anemia (27.8% vs. 18.6%, *p* < .001), and cancer (4.8% vs. 3.4%, *p* < .001). After performing propensity score match, a total of 31 862 patients were matched within each group. COVID‐AF cohort had higher inpatient mortality (22.2% vs. 15.3%, *p* < .001) and more complications, mainly including cardiac arrest (3.9% vs. 2.3%, *p* < .001), cardiogenic shock (0.9% vs. 0.3%, *p* < .001), hemorrhagic stroke (0.4% vs. 0.3%, *p* = .025), and ischemic stroke (1.3% vs. 0.7%, *p* < .001). COVID‐AF cohort was more costly, with a longer length of stay, and a higher total charge.

**Conclusion:**

AF is common in patients hospitalized for COVID‐19, and is associated with poorer in‐hospital mortality, immediate complications and increased healthcare resource utilization.

## Graphical abstract

Atrial fibrillation (AF) is notably prevalent among Coronavirus disease‐2019 (COVID‐19) hospitalized patients, correlating with higher in‐hospital mortality, immediate complications, and an increase in healthcare resource demands.

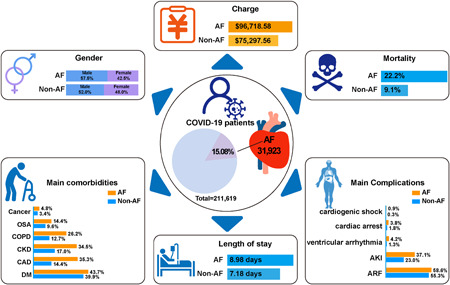

## 1 INTRODUCTION

The Coronavirus disease‐2019 (COVID‐19) pandemic has posed a challenge to healthcare systems worldwide.^1^, ^2^ As researchers continue to understand the intricate interactions between COVID‐19 and various pre‐existing medical conditions, emerging evidence suggests that cardiovascular comorbidities may play a significant role in the clinical course and outcomes of COVID‐19 patients.^3^, ^4^

Among these comorbidities, atrial fibrillation (AF), one of the most common cardiac arrhythmias,^5^ has emerged as a topic of interest in COVID‐19 research. An increasing body of evidence suggests that AF, may play a crucial role in shaping the clinical outcomes of COVID‐19 patients.^6^ Several preliminary studies have hinted at a potential bidirectional link between COVID‐19 and AF. One study conducted by Daniel D et al. reported a higher prevalence of AF in severe COVID‐19 cases compared to milder cases.^1^ Additionally, recent evidence suggests that COVID‐19 may lead to myocardial injury and contribute to AF initiation or exacerbation.^2^ However, the mechanisms underlying this relationship remain largely elusive.

Understanding the impact of AF on COVID‐19 patients is essential for optimizing clinical management strategies. The presence of AF may pose unique challenges for antiviral therapies, immunomodulatory drugs, and anticoagulant regimens used in COVID‐19 treatment. This research paper aims to comprehensively investigate the implications of AF on hospitalized COVID‐19 patients.

## 2 METHODS

### 2.1 Ethical statement

In our study, the National Inpatient Sample (NIS) 2020 database was used to examine the association of COVID‐19 and AF in‐hospital outcomes. As NIS data is deidentified and publicly available, this study was exempt from institutional review board evaluation.

### 2.2 Data

The NIS 2020 database was used to examine the association of COVID‐19 and AF in‐hospital outcomes. NIS is a large, publicly available, all‐payer inpatient healthcare database maintained by the Agency for Healthcare Research and Quality in the United States, representing an approximately 20% stratified sample of discharges from community hospitals and approximately 95% of the US population.^7^ COVID‐19 diagnosis was identified as code “U071 (2019 novel coronavirus disease)” by the International Classification of Diseases, 10th edition, Clinical Modification (ICD‐10‐CM).

### 2.3 Study population

Patients with a primary diagnosis of COVID‐19 and with discharge status were included in the study. Patient demographics was extracted for data analysis; these include age, sex, race, geographic location, household income, primary payer, hospital type, region and bed size, and common cardiovascular comorbidities (reported history of smoking, obesity, anxiety, depression, hypertension, hyperlipidemia, diabetes mellitus [DM], obstructive sleep apnea [OSA] syndrome, chronic obstructive pulmonary diseases [COPD], coronary artery disease [CAD], chronic kidney disease [CKD]).^8^, ^9^, ^10^, ^11^, ^12^ The selected patients were divided into two groups: those with AF and those without.

### 2.4 Outcomes

The primary endpoints of the studies were (1) in‐hospital mortality, (2) in‐hospital complications, which include, cardiac arrest, cardiogenic shock, ventricular arrhythmia, acute respiratory failure (ARF), acute kidney injury (AKI), hemorrhagic stroke, and ischemic stroke; (3) length of stay (LOS) and total cost during hospitalization. Of note, those outcomes were identified by ICD‐10 codes within NIS database (Supporting Information: Table 1).

### 2.5 Statistical analysis

Mean and standard deviation (SD) were used to characterize continuous variables. Percentage was used to describe categorical variables. *T* test was used to compare continuous variables, and *χ* ^2^ was used for categorical variables. A *p*‐value less than .05 was considered statistically significant. Statistical analysis was conducted using the R statistical software (version 3.6.1; R Foundation for Statistical Computing).

We first analyzed the overall characteristics of the COVID‐AF and COVID‐non‐AF cohort, and compared the in‐hospital outcomes of two cohorts. We then used propensity score matching method to reduce selection bias with a 1:1 target ratio from the two cohorts. Multivariate logistic regression model was used to adjust patient demographics (age, sex, race, geographic location, household income, and primary payer), hospital demographics (hospital type, region, and bed size), and common cardiovascular comorbidities as mentioned above. Finally, we compared in‐hospital outcomes between the two cohorts before and after matching to demonstrate the impact of AF on in‐hospital outcomes of COVID‐19 patients.

## 3 RESULTS

### 3.1 Baseline characteristics

Among 211 619 patients with a primary diagnosis of COVID‐19, 31 923 (15.08%) had a secondary diagnosis of AF. Detailed baseline characteristics were described in Table 1.

**Table 1:** Baseline characteristics.

Variables	Unmatched cohort			Propensity‐matched cohort		
	COVID‐19 without AF	COVID‐19 with AF	*p* Value	COVID‐19 without AF	COVID‐19 with AF	*p* Value
*n*	179 696	31 923		31 862	31 862	
Age, mean (SD)	62.21 (16.84)	75.83 (11.25)	<.001	75.97 (11.14)	75.81 (11.25)	.073
Sex, *n* (%)			<.001			.586
Male	93 384 (52.0)	18 361 (57.5)		18 273 (57.4)	18 306 (57.5)	
Female	86 304 (48.0)	13 562 (42.5)		13 589 (42.6)	13 556 (42.5)	
Unknown	8 (0.0)	0 (0.0)		0 (0.0)	0 (0.0)	
Race, *n* (%)			<.001			.82
White	85 351 (47.5)	22 281 (69.8)		22 166 (69.6)	22 222 (69.7)	
Black	34 013 (18.9)	3921 (12.3)		4047 (12.7)	3921 (12.3)	
Hispanic	39 340 (21.9)	3096 (9.7)		3026 (9.5)	3096 (9.7)	
Asian/Pacific Islander	5925 (3.3)	687 (2.2)		686 (2.2)	687 (2.2)	
Native American	1959 (1.1)	172 (0.5)		169 (0.5)	172 (0.5)	
Other	7609 (4.2)	820 (2.6)		818 (2.6)	820 (2.6)	
Unknown	5499 (3.1)	946 (3.0)		950 (3.0)	944 (3.0)	
Patient location, *n* (%)			<.001			.771
“Central” counties of metro areas of ≥1 million population	58 330 (32.5)	8492 (26.6)		8548 (26.8)	8483 (26.6)	
“Finge” counties of metro areas of ≥1 million population	41 451 (23.1)	7727 (24.2)		7596 (23.8)	7707 (24.2)	
Counties in metro areas of 250 000–999 999 population	33 487 (18.6)	6041 (18.9)		6043 (19.0)	6032 (18.9)	
Counties in metro areas of 50 000–249 999 population	15 511 (8.6)	3241 (10.2)		3155 (9.9)	3231 (10.1)	
Micropolitan counties	16 683 (9.3)	3387 (10.6)		3473 (10.9)	3383 (10.6)	
Nonmetropolitan or micropolitan counties	13 483 (7.5)	2955 (9.3)		2963 (9.3)	2946 (9.2)	
NA	751 (0.4)	80 (0.3)		84 (0.3)	80 (0.3)	
Mean household income, *n* (%)			<.001			.48
$1–$42 999	61 732 (34.4)	9653 (30.2)		9786 (30.7)	9640 (30.3)	
$43 000–$53 999	48 412 (26.9)	9193 (28.8)		9108 (28.6)	9172 (28.8)	
$54 000–$70 999	38 604 (21.5)	7026 (22.0)		6954 (21.8)	7015 (22.0)	
$71 000 or more	28 071 (15.6)	5612 (17.6)		5539 (17.4)	5598 (17.6)	
Unknown	2877 (1.6)	439 (1.4)		475 (1.5)	437 (1.4)	
Primary payer, *n* (%)			<.001			.341
Medicare	84 580 (47.1)	25 064 (78.5)		25 249 (79.2)	25 007 (78.5)	
Medicaid	24 029 (13.4)	1475 (4.6)		1425 (4.5)	1475 (4.6)	
Private including HMO	54 375 (30.3)	4145 (13.0)		4035 (12.7)	4142 (13.0)	
Self‐pay	6859 (3.8)	331 (1.0)		300 (0.9)	331 (1.0)	
No charge	486 (0.3)	35 (0.1)		30 (0.1)	35 (0.1)	
Other	9008 (5.0)	825 (2.6)		776 (2.4)	824 (2.6)	
Unknown	359 (0.2)	48 (0.2)		47 (0.1)	48 (0.2)	
Hospital type, *n* (%)			<.001			.772
Rural	20 528 (11.4)	4137 (13.0)		4185 (13.1)	4131 (13.0)	
Urban nonteaching	34 343 (19.1)	6243 (19.6)		6249 (19.6)	6228 (19.5)	
Urban teaching	124 825 (69.5)	21 543 (67.5)		21 428 (67.3)	21 503 (67.5)	
Hospital region, *n* (%)			<.001			.203
Northeast	31 548 (17.6)	5853 (18.3)		5852 (18.4)	5840 (18.3)	
Midwest	40 151 (22.3)	9062 (28.4)		8814 (27.7)	9036 (28.4)	
South	76 105 (42.4)	12 448 (39.0)		12 643 (39.7)	12 429 (39.0)	
West	31 892 (17.7)	4560 (14.3)		4553 (14.3)	4557 (14.3)	
Hospital bed size, *n* (%)			.25			.97
Small	46 135 (25.7)	8103 (25.4)		8062 (25.3)	8085 (25.4)	
Medium	51 753 (28.8)	9332 (29.2)		9336 (29.3)	9313 (29.2)	
Large	81 808 (45.5)	14 488 (45.4)		14 464 (45.4)	14 464 (45.4)	
Comorbidities, *n* (%)						
Smoking	46 302 (25.8)	10 334 (32.4)	<.001	10 378 (32.6)	10 307 (32.3)	.554
Hypertension	77 634 (43.2)	10 421 (32.6)	<.001	10 943 (34.3)	10 421 (32.7)	<.001
DM	71 647 (39.9)	13 964 (43.7)	<.001	14 136 (44.4)	13 938 (43.7)	.116
Hyperlipidemia	71 446 (39.8)	17 443 (54.6)	<.001	17 656 (55.4)	17 400 (54.6)	.042
Obesity	52 227 (29.1)	7678 (24.1)	<.001	7725 (24.2)	7664 (24.1)	.579
Anxiety	22 852 (12.7)	4197 (13.1)	.035	4199 (13.2)	4192 (13.2)	.944
Depression	20 040 (11.2)	4061 (12.7)	<.001	4065 (12.8)	4058 (12.7)	.943
OSA	17 166 (9.6)	4597 (14.4)	<.001	4450 (14.0)	4571 (14.3)	.173
CKD	30 601 (17.0)	11 011 (34.5)	<.001	10 903 (34.2)	10 966 (34.4)	.605
COPD	22 758 (12.7)	8349 (26.2)	<.001	8209 (25.8)	8313 (26.1)	.352
CAD	25 789 (14.4)	11 273 (35.3)	<.001	10 967 (34.4)	11 213 (35.2)	.042
Anemia	33 377 (18.6)	8859 (27.8)	<.001	8684 (27.3)	8824 (27.7)	.217
Cancer	6033 (3.4)	1539 (4.8)	<.001	1528 (4.8)	1537 (4.8)	.882

Abbreviations: AF, atrial fibrillation; CAD, coronary artery disease; CKD, chronic kidney disease; COPD, chronic obstructive pulmonary disease; COVID‐19: Coronavirus disease‐2019; DM, diabetes mellitus; OSA, obstructive sleep apnea.

Before propensity score match, COVID‐AF cohort consists of older patients (75.8 vs. 62.2‐year‐old, *p* < .001) with a higher proportion of men (57.5% vs. 52.0%, *p* < .001). This group was associated with a higher reported smoking history (32.4% vs. 25.8%, *p* < .001), DM (43.7% vs. 39.9%, *p* < .001), hyperlipidemia (54.6% vs. 39.8%, *p* < .001), CKD (34.5% vs. 17.0%, *p* < .001), COPD (26.2% vs. 12.7%, *p* < .001), CAD (35.3% vs. 14.4%, *p* < .001), anxiety (13.1% vs. 12.7%, *p* < .035), depression (12.7% vs. 11.2%, *p* < .001), OSA (14.4% vs. 9.6%, *p* < .001), anemia (27.8% vs. 18.6%, *p* < .001), and cancer (4.8% vs. 3.4%, *p* < .001). Separately, the COVID‐non‐AF group showed higher proportions of hypertension and obesity. Before matching, the two groups exhibited disparities in various socioeconomic factors, including race, patient location, primary payer, hospital type, and region.

**Figure 1:**
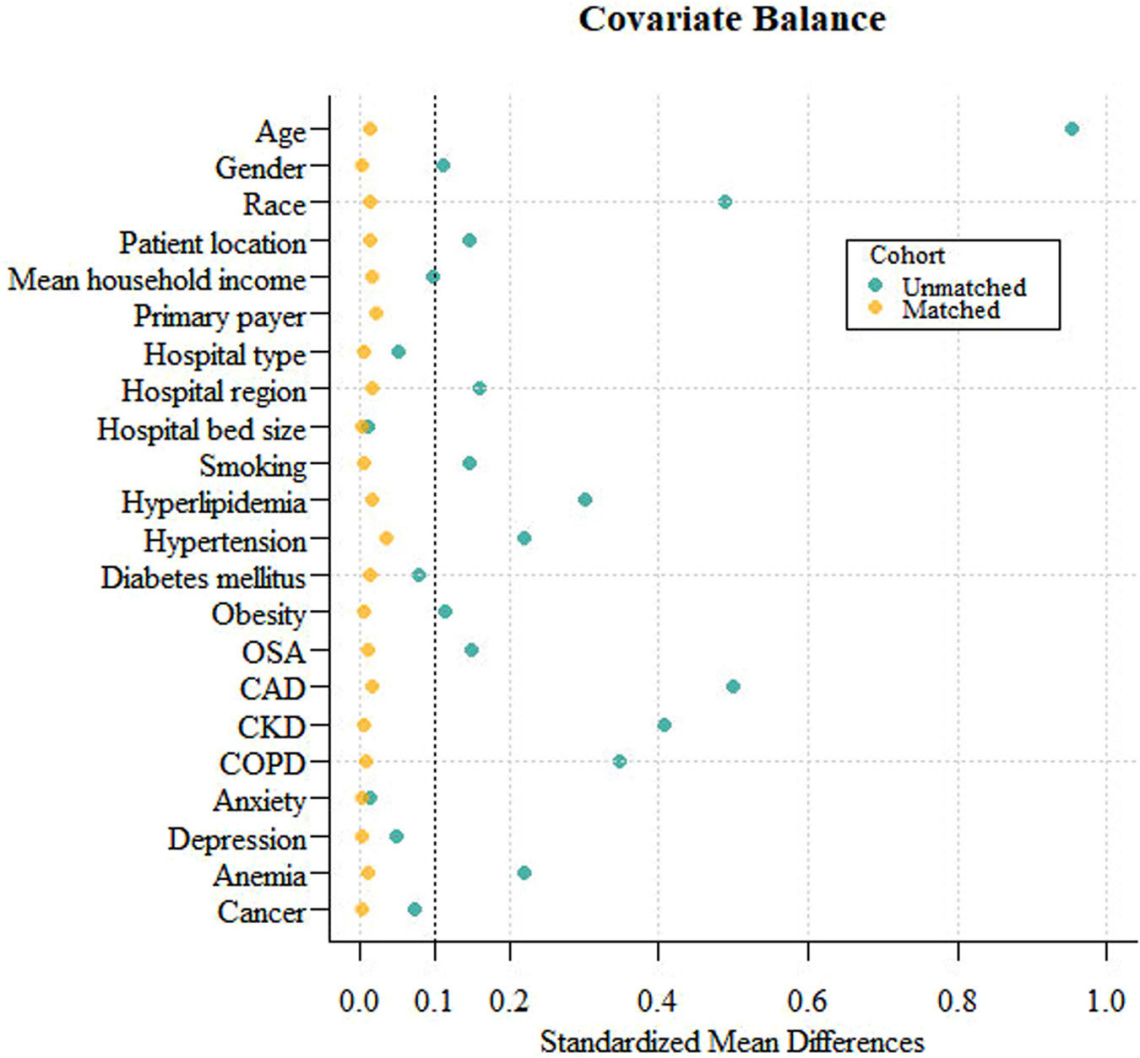
Standardized mean differences of covariates before and after propensity score matching between COVID‐19 patients with and without atrial fibrillation. Standardized mean difference was used to examine the balance of the covariate distribution between the matched atrial fibrillation and the nonatrial fibrillation groups. All standardized mean differences of covariate distributions in this study were less than .1, which was considered balanced. CAD, coronary artery disease; CKD indicates chronic kidney disease; COPD, chronic obstructive pulmonary disease; COVID‐19, Coronavirus disease‐2019; OSA, obstructive sleep apnea.

Following the propensity score match, the COVID‐AF (*n* = 31 862) and COVID‐non‐AF (*n* = 31 862) groups exhibited well‐matched baseline characteristics (*p* > .05). All baseline characteristics included had standard mean differences of less than .1 between the two cohorts (Figure 1).

### 3.2 In‐hospital mortality and complications

**Figure 2:**
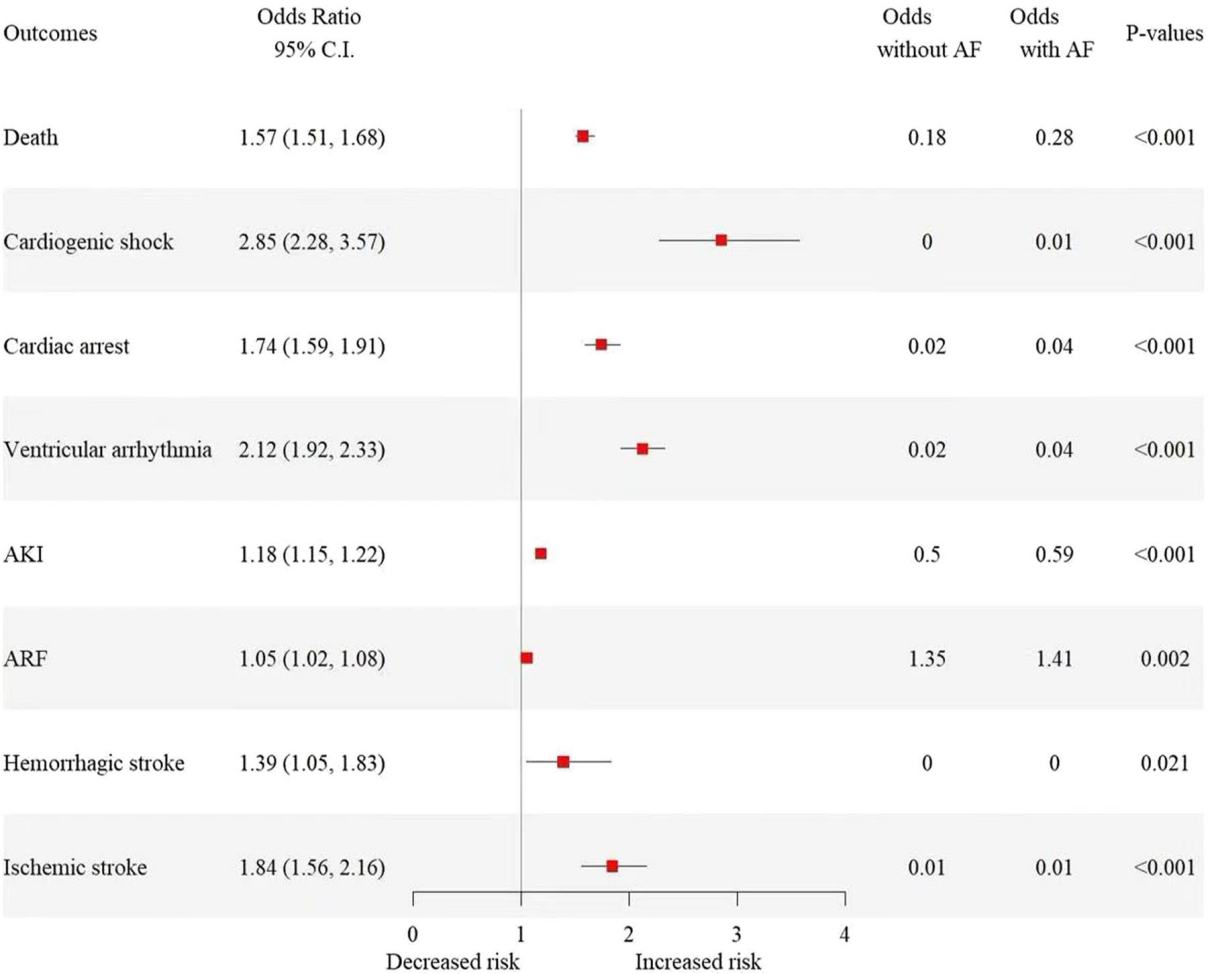
Forest plot graph showing adjusted odds ratio for in‐hospital outcomes after propensity score matching. AF, atrial fibrillation; AKI, acute kidney injury; ARF, acute respiratory failure.

The in‐hospital mortality rate in COVID‐AF cohort was higher with and without propensity score match (22.2% vs. 15.3%; 22.2% vs. 9.1%, both *p* < .001). The COVID‐AF cohort had a higher rate of overall complications before propensity score match, including cardiac arrest (3.8% vs. 1.8%, *p* < .001), cardiogenic shock (0.9% vs. 0.3%, *p* < .001), ventricular arrhythmia (4.2% vs. 1.3%, *p* < .001), ARF (58.6% vs. 55.3%, *p* < .001), AKI (37.1% vs. 23.0%, *p* < .001), ischemic stroke (1.3% vs. 0.6%, *p* < .001), and hemorrhagic stroke (0.4% vs. 0.2%, *p* < .001) (Table 2). The difference persists after propensity score match, where the COVID‐AF cohort continue to show a higher rate of complications above (all *p* < .05, Table 2). The adjusted odds ratios for in‐hospital complications after the propensity score match is illustrated in Figure 2.

**Table 2:** In‐hospital outcomes.

Variables	Unmatched cohort			Propensity‐matched cohort		
	COVID‐19 without AF	COVID‐19 with AF	*p* Value	COVID‐19 without AF	COVID‐19 with AF	*p* Value
*n*	179 696	31 923		31 862	31 862	
Outcomes						
Death, *n* (%)	16 383 (9.1)	7073 (22.2)	<.001	4883 (15.3)	7061 (22.2)	<.001
Cardiac arrest, *n* (%)	3257 (1.8)	1229 (3.8)	<.001	717 (2.3)	1228 (3.9)	<.001
Cardiogenic shock, *n* (%)	545 (0.3)	295 (0.9)	<.001	104 (0.3)	295 (0.9)	<.001
Ventricular arrhythmia, *n* (%)	2344 (1.3)	1354 (4.2)	<.001	651 (2.0)	1347 (4.2)	<.001
AKI, *n* (%)	41 291 (23.0)	11 854 (37.1)	<.001	10 598 (33.3)	11 823 (37.1)	<.001
ARF, *n* (%)	99 355 (55.3)	18 698 (58.6)	<.001	18 276 (57.4)	18 660 (58.6)	.002
Hemorrhagic stroke, *n* (%)	414 (0.2)	119 (0.4)	<.001	86 (0.3)	119 (0.4)	.025
Ischemic stroke, *n* (%)	1094 (0.6)	417 (1.3)	<.001	228 (0.7)	417 (1.3)	<.001
LOS, mean (SD)	7.18 (7.86)	8.98 (9.06)	<.001	7.81 (7.70)	8.98 (9.06)	<.001
Total charge, mean (SD)	75 297.56 (147 365.86)	96 718.58 (161 781.41)	<.001	74 139.28 (120 951.25)	96 731.19 (161 823.99)	<.001

Abbreviations: AF, atrial fibrillation; AKI, acute kidney failure; ARF, acute respiratory failure; COVID‐19: Coronavirus disease‐2019; LOS, length of stay.

### 3.3 LOS and total cost

The COVID‐AF cohort had longer LOS (9.0 ± 9.1 days vs. 7.2 ± 7.9 days, *p* < .001), and higher total cost (96 718.6 ± 161 781.4 vs. 75 297.56 ± 147 365.8 dollars, *p* < .001) compared to the COVID‐non‐AF cohort. The difference again persists after propensity score match (*p* < .005) (Table 2).

## 4 DISCUSSION

In this study, we conducted a real‐world analysis to assess the prevalence and features of patients admitted for COVID‐19 infection with concurrent diagnosis of AF, and described its correlation of higher mortality and worse outcomes. Among the 211 619 patients diagnosed with COVID‐19, 15.1% had a secondary diagnosis of AF, indicating that AF is not a rare comorbidity in COVID‐19 patients. The COVID‐AF cohort also exhibited increased mortality and higher incidences of major cardiovascular and cerebral complications, including cardiac arrest, cardiogenic shock, ventricular arrhythmia, ARF, AKI, ischemic stroke, and hemorrhagic stroke. These findings suggest that AF may serve as an indicator of the severity of COVID‐19, rather than merely being a concurrent condition. COVID‐AF group also utilized more health care resources, with longer LOS and higher total cost.

Demographically, patients in COVID‐AF cohort are older with more comorbidities. This observation is consistent with prior research that AF is associated with increased age.^13^ The age correlation can be attributed to age‐related changes in the atria. With aging, there are increased cardiac tissue fibrosis, and alterations in ion channel function that predispose older individuals to arrhythmia development.^14^ Older age is also associated with a higher burden of comorbidities, which further increase individuals' vulnerability to COVID infection and AF complications. Separately, COVID‐AF cohort has a higher proportion of men. Being men is also associated with an increased risk of both AF^15^ and severe COVID‐19.^16^ Hormonal, genetic, and immunological factors are thought to play a role in this gender‐specific difference in the disease susceptibility and progression.^17^, ^18^

Patients in COVID‐AF cohort has a higher rate of cardiovascular comorbidities. For example, the association between AF and DM as well as hyperlipidemia has been extensively reported.^19^ DM and hyperlipidemia contribute to atrial remodeling and pro‐inflammatory changes in the atrial tissue, fostering the development and persistence of AF.^20^, ^21^ Additionally, CKD has also been found related to a higher incidence of AF due to fluid‐electrolyte imbalances, inflammation, and oxidative stress.^22^ CKD is an independent predictor of severe outcomes in COVID‐19 patients, contributing to the higher rate of mortality and complications in COVID‐AF group.^13^ AF is prevalent in COPD. The systemic inflammation and oxidative stress in COPD, along with respiratory distress, can contribute to atrial remodeling, thereby increasing the likelihood for AF development.^23^ Overall, the presence of AF in COVID‐19 patients may be an indicator of a patient's overall wellbeing and comorbidity burden.

It is therefore not surprising that we found that COVID‐AF cohort has significantly higher in‐hospital mortality and complication rate, longer LOS, and increased healthcare costs compared to COVID‐non‐AF cohort. The worse outcomes persist after propensity score matching, adjusting for the comorbidities and baseline characteristics of both groups. Our result indicates that AF poses an independent risk for adverse outcomes in COVID‐19 patients. The mechanisms underlying this association are probably multifactorial. First, COVID‐19 triggers an exaggerated immune response, leading to a cytokine storm, which contributes to organ damage and exacerbates the disease severity.^24^, ^25^, ^26^ AF can not only be precipitated by systemic inflammation and immune dysregulation but also exacerbates these conditions.^27^, ^28^, ^29^ In COVID patients with AF, the inflammation may be further intensified, leading to a dysregulated immune response, increased vascular permeability, and coagulopathy and contributing to the observed worse outcomes. Second, AF‐related hemodynamic changes, including rapid ventricular rates and loss of atrial contraction, reduce cardiac output and impair cardiac reserve. In the context of respiratory distress and hypoxia with COVID pneumonia, the reduced cardiac reserve in AF patients may exacerbate hypoxemia and organ damage, potentially leading to a higher risk of acute respiratory distress syndrome and other complications.^30^, ^31^, ^32^, ^33^ Third, COVID‐19 is associated with endothelial dysfunction and thrombosis,^34^ which can cause microvascular and macrovascular complications. AF is also known to promote a prothrombotic state,^35^ and the combination of COVID‐19 and AF may result in a synergistic increase in thrombotic events^36^ and adverse outcomes.^37^ Anticoagulation therapy had been suggested in AF patients with high risk of thrombosis.^38^ The current recommendation is also in favor of prophylactic‐intensity anticoagulation for patients with COVID‐19‐related critical illness.^39^ The thrombotic state and the emphasis on anticoagulation therapy can perhaps explain the increased incidence of cerebrovascular events in COVID‐AF cohort.

It is plausible that the coexistence of AF is a indicator of a state of increased physiological stress, potentially resulting in a more severe hospital course in patients admitted for COVID infection.^21^ The clinical implication of such findings in COVID‐AF cohort warrants further study to improve outcome and reduce complications. Identifying COVID‐19 patients with a comorbidity of AF early in their hospitalization allows clinicians to recognize those at higher risk for severe outcomes. Efforts to optimize AF before and during COVID‐19 infection might positively influence patient outcomes.

## 5 LIMITATIONS

Several limitations should be acknowledged. First, the study's retrospective nature introduces inherent biases and potential unaccounted confounders. Such can influence the observed associations between AF and COVID‐19 outcomes. Second, the study focused on hospitalized patients, which may not fully represent the entire COVID‐19 infected population in the community. Third, the study lacked data on the timing of AF onset in relation to the COVID‐19 infection, which limits the ability to further understand the intricate relationship between the two conditions. Last, we did not note or compare the treatment strategies and their impact on outcome between two cohorts due to the nature of the NIS database. Further research is needed to assess the impact of therapies on COVID‐19 outcomes in patients with AF.

## 6 CONCLUSION

Our study underscores the significant impact of AF on the outcomes of hospitalized COVID‐19 patients. The findings of the study suggest that COVID‐19 patients with AF constitute a vulnerable subgroup with worse clinical outcomes. Further research is warranted to enhance our understanding of the intricate relationship between AF and COVID‐19 and develop evidence‐based strategies to mitigate its adverse effects on this high‐risk subgroup.

## AUTHOR CONTRIBUTIONS

Haiming Niu, Pengyang Li, Jianwei Li, and Yong Yuan conceived the study. Haiming Niu, Jianwei Li, Pengyang Li, Catherine Teng, Xiaojia Lu, Chengyue Jin, Ao Shi, Miaolian Chen, Xiaoqing Shen, and Qiqi Chen were involved in the data collection process. Pengyang Li and Peng Cai analyzed and interpreted the data. Pengyang Li, Yong Yuan, Catherine Teng, Peng Cai, Miaolian Chen, Ao Shi, Xiaoqing Shen, and Qiqi Chen interpreted the results. Haiming Niu, Pengyang Li, Catherine Teng, Xiaojia Lu, Chengyue Jin, and Xiaoqing Shen wrote the manuscript. All authors revised the manuscript and approved the final manuscript for submission.

## CONFLICT OF INTEREST STATEMENT

The authors declare no conflict of interest.

## Supporting information

Supporting information.

## Data Availability

This study is conducted using data from the 2020 National Inpatient Sample (NIS) database, which is accessible at https://hcup-us.ahrq.gov/nisoverview.jsp.
