# Supplementary material for: The incidence and impact of atrial fibrillation on hospitalized Coronavirus disease‐2019 patients
Source: Clin Cardiol. 2024 Feb 25;47(2):e24240. doi: 10.1002/clc.24240 (PMC10894524; doi:10.1002/clc.24240)
Supplement: Supplementary file 1 — Supporting information. [file CLC-47-e24240-s001.doc]

Supplemental Table 1 International Classification of Disease, 10th edition, Clinical Modification (ICD-10-CM) Codes Used for Comorbidities and Complications

| **Comorbidities/Complications** | **ICD-10 Code** |
| --- | --- |
| AF  COVID-19 | I48  U071 |
| **Comorbidities** |  |
| CAD | I251, I255, I257, I258, I259 |
| Smoking | F17-F17299, Z720, Z87891 |
| Hypertension | I10, I15-I159 |
| Diabetes mellitus | E10-E109, E11-E119, E13-E139 |
| Hyperlipidemia | E780-E785 |
| Obesity | Z683-Z6839, Z684-Z6845, E66-E669 |
| Anxiety | F064, F40-F409, F41-F419 |
| Depression | F32-F329, F33-F339, F341 |
| OSA | G473-G4739 |
| CKD | N18-N189 |
| COPD | J41-J418, J42, J43-J439, J44-J449 |
| Anemia | D50-D59, D60-D64 |
| Cancer | C00-C969, D00-D099 |
| **Complications** |  |
| Cardiac arrest | I46-I469 |
| Cardiogenic shock | R570 |
| Ventricular arrhythmia | I472, I490-I4902 |
| Acute kidney injury | N17-N179 |
| Acute respiratory failure | J960-J9622 |
| Hemorrhagic stroke | I60-I62 |
| Ischemic stroke | I63 |

AF, atrial fibrillation; CAD, coronary artery disease; CKD, chronic kidney disease; COPD, chronic obstructive pulmonary disease; COVID-19, Coronavirus disease 2019; OSA, obstructive sleep apnea;
